# HIV-1 did not contribute to the 2019-nCoV genome

**DOI:** 10.1080/22221751.2020.1727299

**Published:** 2020-02-14

**Authors:** Chuan Xiao, Xiaojun Li, Shuying Liu, Yongming Sang, Shou-Jiang Gao, Feng Gao

**Affiliations:** a Department of Chemistry and Biochemistry, The University of Texas at El Paso, El Paso, TX, USA; b Department of Medicine, Duke University Medical Center, Durham, NC, USA; c NA BioTech Corp, M2D2 Incubator, University of Massachusetts Medical School, Worcester, MA, USA; d Department of Agricultural and Environmental Sciences, Tennessee State University, Nashville, TN, USA; e UPMC Hillman Cancer Center, Department of Microbiology and Molecular Genetics, University of Pittsburgh, Pittsburgh, PA, USA; f National Engineering Laboratory for AIDS Vaccine, School of Life Sciences, Jilin University, Changchun, People’s Republic of China

When a new pathogen that causes a global epidemic in humans, one key question is where it comes from. This is especially important for a zoonotic infectious disease that jumps from animals to humans. Knowing the origin of such a pathogen is critical to develop means to block further transmission and to develop vaccines. Discovery of the origin of a newly human pathogen is a sophisticated process that requires extensive and vigorous scientific validations and generally takes many years, such as the cases for HIV-1 [1], SARS [2] and MERS [3]. Unfortunately, before the natural sources of new pathogens are clearly defined, conspiracy theories that the new pathogens are man-made often surface as the source. However, in all cases, such theories have been debunked in history.

Infection from an emerging pathogenic coronavirus was first reported in December 2019 in China. It has now affected over 42,000 people and caused over 1,000 deaths in 25 countries (https://2019ncov.Chinacdc.Cn/2019-Ncov). The complete genome of this new virus was quickly sequenced and made public on January 12, only about 2 weeks after the disease was first observed [4]. It was named as 2019-nCoV the following day by the World Health Organization (WHO). Phylogenetic analysis shows that 2019-nCoV is a new member of coronaviruses that infect humans. It is genetically homogenous but distinct from coronaviruses that cause SARS and MERS [5,6]. However, it shares a high level of genetic similarity (96.3%) with a bat coronavirus RaTG13 which was obtained from bat in Yunnan in 2013, suggesting that RaTG13-like viruses are most likely the reservoir, but not the immediate sources of the current 2019-nCoV viruses [7].

Lack of the definite origin of 2019-nCoV has led to speculation that 2019-nCoV might be derived from genetic manipulation or even for the purpose of use as a bioweapon. This notion has been fully debunked in the media. A recent informally presented report, however, showed that 2019-nCoV had four insertions in the spike glycoprotein gene that is critical for the virus to enter the target cells when compared to other coronaviruses [8]. It was claimed that these inserts were either identical or similar to the motifs in the highly variable (V) regions (V1, V4 and V5) in the envelope glycoprotein or in the Gag protein of some unique HIV-1 strains from three different countries (Thailand, Kenya and India). Together with the structure modelling analysis, the authors speculated that these motif insertions sharing similarity with HIV-1 proteins could provide an enhanced affinity towards host cell receptors and increase the range of host cells of 2019-nCoV. This study implies that 2019-nCoV might be generated by gaining gene fragments from the HIV-1 genome.

Current report conducted careful examination of the sequences of 2019-nCoV, other CoV viruses and HIV-1 as well as GenBank database. Our results demonstrated no evidence that the sequences of these four inserts are HIV-1 specific or the 2019-nCoV viruses obtain these insertions from HIV-1. First, the results of blast search of these motifs against GenBank shows that the top 100 identical or highly homologous hits are all from host genes of mammalian, insects, bacterial and others. There are only a few hits on coronaviruses, but none of them are HIV-1 related. Blast against viral sequence database also showed these insertion sequences widely exist in all kinds of viruses from bacteriophage, influenza, to giant eukaryotic viruses (Table 1). More hits were found for coronaviruses and a few also hit on HIV-1 sequences than the search against the entire database (Table 1). However, while the 100% match between the insertion 1 and 2 sequences and the HIV sequences were found in 19 entries, the matches between the insertion 3 and 4 sequences and HIV-1 sequences were rather poor (from 42% to 88%). Moreover, the insertion 4 sequence ambiguously hit multiple different genes (*gag, pol* and *env*) in the HIV-1 genome, suggesting that similarities (as low as 42%) between them are too low to be reliable. Search these four insertion sequences against HIV-1 Sequence Database (https://www.hiv.lanl.gov/components/sequence/HIV/search/search.html) yielded similar results. Sequences that completely match the insertion 3 and 4 sequences were not found in any HIV-1 sequences. This clearly shows that these insertioin sequences are widely present in living organisms including viruses, but not HIV-1 specific. All these regions in HIV-1 envelope glycoprotein are highly variable with many large insertions and deletions, indicating that they are not essential for biological functions of HIV-1 envelope glycoprotein. The detection of completely matched sequences of 1 and 2 insertions in only a few HIV-1 strains demonstrated that four insertions are very rare or not present among tens of thousands of natural HIV-1 sequences. This also explains why four insertion homolog sequences could only be independently found in different HIV-1 genomes [8]. Because of their poor identities to and rareness in the HIV-1 sequences, HIV-1 could not be the source for those insertion sequences in the 2019-nCoV genome.

**Table 1.** Blast search results of four insertion sequences against sequence databases.

Database	Gene source	Insertion 1 TNGTKR	Insertion 2 HKNNKS	Insertion 3 RSYLTPGDSSSG	Insertion 4 QTNSPRRA
Whole database	CoV	2 (2)	0	3 (3)	2 (2)
	HIV-1	0	0	0	0
	Prokaryotic	27 (27)	3 (3)	74 (0)	66 (1)
	Eukaryotic	71 (71)	97 (97)	23 (0)	32 (1)
Only viral database	CoV	3 (3)	3 (3)	5 (3)	3 (2)
	HIV-1	18 (18)	1 (1)	4 (0)*	6 (0)**
	Other Eukaryotic viruses	49 (2)	66 (8)	69 (0)	62 (0)
	Prokaryotic viruses	29 (13)	30 (1)	21 (0)	28 (0)
	Unclassified virus	1 (1)	0	1 (0)	1 (0)

Top 100 hits are analyzed and the numbers of 100% matches are shown in parentheses. * Similarity at 67%; ** Random hits in Gag, Pro and Env sequences with similarity between 42% and 88%.

**Figure 1.**
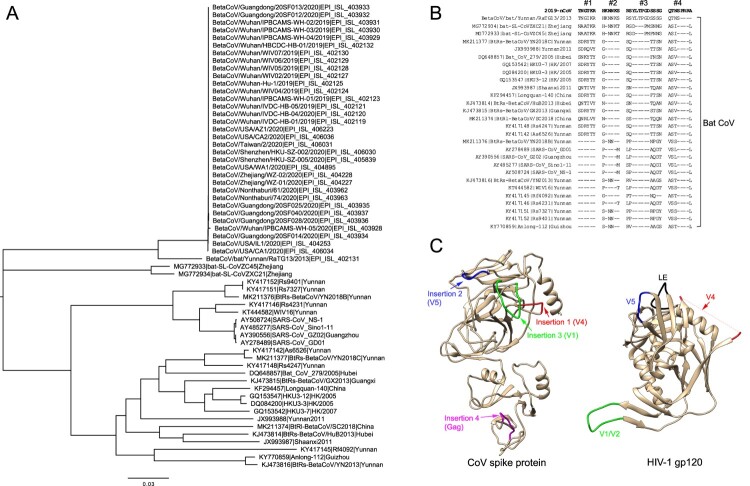
Sequence and structure analysis of 2019-nCoV and bat coronaviruses. (A) Phylogenetic tree analysis of the spike gene sequences. (B) Sequence alignment of suspected insertion sites between the 2019-nCoV and bat coronavirus sequences. The deletions in the alignment are shown as dashes. The numbers of insertions are indicated at the top of the alignment. (C) Structure comparison of the four insertions in the CoV spike protein and HIV-1 gp120. 2019-nCoV structure was modelled using I-TASSER server with default parameters. Only relevant domains with residues 1 to 708 (exclude residues from 305 to 603) were presented as ribbon diagram. The four insertions were labelled and coloured in red, blue, green and magenta, respectively. HIV-1 gp120 structure (PDB 1GC1) is presented as ribbon diagram. V4, V5, V1/V2 and LE loops were labelled and coloured in red, blue, green, and black, respectively.

Second, these insertions are present not only in 2019-nCoV viruses but also in three betaCoV sequences from bats: two (ZC45 and ZXC21) from Zhejiang deposited in GenBank in 2018 and RaTG13 from Yunnan obtained in 2013 [8]. The RaTG13 is much more similar to 2019-nCoV than both ZC45 and ZXC21 (Figure 1A). The similarity of the spike protein between RaTG13 and 2019-nCoV is 97.7%. In the RaTG13 genome, two inserts are identical (HKNNKS and RSYLTPGDSSSG) to those in 2019-nCoV, one has one T → I substitution (TNG*I*KR), and the fourth one misses the C-terminal 4 amino acids (QTNS*----*) (Figure 1B). ZC45 and ZXC21 are more divergent from 2019-nCoV than RaTG13, but both also contain similar insertions at three insertion sites, except insertion 4 (Figure 1B). Furthermore, many other CoV viruses have similar insertions but with different sequences at the insertion 1 position. These results clearly show that three out of four of these inserts naturally exist in three bat CoV viruses before 2019-nCoV was identified. This undoubtedly refutes the possibility that 2019-nCoV is generated through obtaining gene fragments from the HIV-1 genome. Instead, it is much more likely that 2019-nCoV originated from RaTG13-like CoV viruses.

Third, insertions 1 and 2 in 2019-nCoV have 6-AA motifs identical to those in V4 and V5 of certain HIV-1 gp120 isolates, which are structurally close to each other but separated by a LE loop (Figure 1C) [9]. However, insertion 3 located between insertions 1 and 2 in 2019-nCoV has sequences similar (with deletions) to those in the V1 region of HIV-1 gp120. V1 is far away from V4 and V5 on the opposite side of gp120, which should not interact with V4/V5 in gp120 (Figure 1C) but is now inserted between V4 and V5 in the modelled the 2019-nCoV spike protein structure [10]. Insertion 4 was found in Gag protein of HIV-1 that is not associated with viral entry. This insertion is located too far to be considered to form the same structural unit with the other three insertions in the 2019-nCoV spike protein (Figure 1C). We do not see any selection benefit or rationale for 2019-nCoV to obtain and mix structurally unrelated parts of HIV-1 to generate a unique structure for its enhanced receptor binding as indicated by the authors [8].

How the three bat CoV viruses obtain those inserts remains unknown. For any virus to obtain additional insert sequences from other organisms, it requires that it has direct interactions with other organisms, most likely through homologous or non-homologous recombination [11]. For bat CoV viruses to gain the gene fragments from HIV-1, it will require both viruses to co-infect the same cells. Because the host cells for bat CoV viruses and HIV-1 are different, the chance for both to exchange genetic materials is negligible. On the contrary, these motifs are widely present in various mammalian cells and so it will be more likely for bat CoV viruses to gain those motifs from the genomes of their infected cells if recombination indeed occurs. However, extensive studies of more CoV viruses in wild and domestic animals are warranted to address this question.

Identification of the origins of these inserted sequences in three bat CoV viruses and the new epidemic 2019-nCoV strain will be important for us to understand how CoV viruses jump from animals to humans and adapt in the latter. Current data showed that RaTG13 is most closely related to 2019-nCoV [7]. However, the genetic difference between them is too high for RaTG13 to serve as the immediate ancestor of 2019-nCoV. Other viruses that are more closely related to 2019-nCoV in intermediate animals like civet for SARS and camel for MERS [3,12] are remained to be identified. More studies are necessary to identify the real source of 2019-nCoV. This may take a long time to identify the origin of 2019-nCoV by screening a large number of wild and domestic animals. In any case, reducing or eliminating direct contacts with wild animals will be critical to control the new epidemic infection diseases in the future.

The advances in bioinformatics analysis tools are widely used to easily and rapidly analyse newly obtained sequences. However, great care is required for comprehensive and thorough analysis to fully understand the real biological implications of the new genomic information. Biased, partial and incorrect analysis can dangerously lead to conclusions that fuel conspiracies and harm the process of true scientific discoveries and the effort to control the damage to public health.
